# Antioxidant and cytoprotective effects of *Nigellasativa* L. seeds on the testis of monosodium glutamate challenged rats

**DOI:** 10.1038/s41598-021-92977-4

**Published:** 2021-06-29

**Authors:** Mahmoud Abd-Elkareem, Mokhless A. M. Abd El-Rahman, Nasser S. Abou Khalil, Ayman S. Amer

**Affiliations:** 1 Department of Anatomy, Histology and Embryology, Faculty of Veterinary Medicine, Assiut University, Assiut, Egypt; 2 Department of Food Science and Technology, Faculty of Agriculture, Assiut University, Assiut, Egypt; 3 Department of Medical Physiology, Faculty of Medicine, Assiut University, Assiut, Egypt; 4 Department of Human Anatomy and Embryology, Faculty of Medicine, Assiut University, Assiut, Egypt

**Keywords:** Cell biology, Physiology, Plant sciences, Structural biology, Anatomy, Medical research

## Abstract

Monosodium glutamate (MSG) is one of the most widely spread food additives that might cause male infertility. However, *Nigella* *sativa* L. seeds (NSS) could provide a solution. This study was designed to investigate the potential effects of NSS on rats ingesting MSG. To achieve this aim, adult male albino rats were randomly equally assigned into three groups for 21 days: control group received no treatment, MSG group received MSG as 30 g/kg feed, and MSG + NSS group received MSG as 30 g/kg and NSS as 30 g/kg feed. Testis histomorphometry showed marked deterioration by MSG as atrophic seminiferous tubules with degeneration of their lining cells, damaged Leydig cells and decreased germ cells number. Periodic Acid Schiff stain indicated irregular interrupted basement membranes. Glutathione reductase, superoxide dismutase 2 (SOD2), and caspase-3 immuno-expressions increased in testicular cells. Testosterone levels were significantly decreased in MSG challenged rats along with significant increase in luteinizing hormone levels, whereas NSS normalized this hormonal profile. MSG exposure also caused significantly increased lipid peroxides (LPO), glutathione-*S*-transferase, and total antioxidant capacity (TAC) whereas nitric oxide and SOD2 were significantly decreased. NSS succeeded in rebalance LPO and TAC and ameliorated the histoarchitectural disturbances. NSS mitigated MSG-induced testicular impairment by its antioxidant and cytoprotective activities.

## Introduction

Monosodium glutamate (MSG), a derivative sodium salt of glutamic acid, is widely consumed as a seasoning and flavoring agent to improve the taste, quality and shelf-life of food products^1^. It is commonly marketed as a safe feed additive without a specific daily intake limit. Its safety as a feed additive remains highly debated. It is generally considered as being harmless by food safety regulatory agencies^2^. Owing to the extensive ability of enterocytes to metabolize glutamate to be a precursor for several other amino acids and as a building block in enterocyte redox potential and detoxification pathways, just a small amount of MSG is absorbed into the blood stream^3,4^. Ingestion of MSG only results in a transient increase in plasma glutamate level, because glutamate is well tolerated by the body through the neutralizing actions of some nutritional substrates intake in concomitant with food-incorporated MSG^5,6^. Nevertheless, presence of high amount of MSG in the processed foods^7^ could predispose to health hazards. Several biological systems are sensitive to the high doses of MSG; however, the reproductive system is considered to be its primary target. This is owing to abundance of glutamate receptors^7^ and polyunsaturated fatty acids and low content of antioxidant reserve^8^ in seminiferous tubules (ST) and spermatozoa, making them more susceptible to its peroxidative and excitatory damage. Plenty of controversies still emerged about its testicular toxicity relative to differences in the age of experimental model, the dose of MSG and the duration of exposure providing a driving force for continuation in exploring this area of research with respect to the multifactorial nature of testicular impairment. Morphological and histomorphometric changes in the testes of adult rats, as well as sperm abnormalities, were reported following intraperitoneal injection of 4 ml MSG/kg BW daily for 14 days. This experimental schedule did not lead to permanent infertility, but the normal morphology of the testis would need a long time to be regained^9^. A dose-dependent differences in the response of reproductive hormone, testicular oxidative status and semen characteristics were noticed in a rat model compromised by MSG indicating that daily oral intake of the higher dose (120 mg/kg BW) for 28 days caused a significant damage to the reproductive performances^7^. Oral supplementation of rats at two age groups with three gradual doses of MSG every 48 h for 6 weeks adversely affected spermatogenesis by disrupting the hypothalamic-pituitary gonadal regulatory axis without direct cyto-toxicological effect on the testis^10^. Varying low doses (0.25, 0.5 or 1 g/kg BW) of MSG were administered orally or subcutaneously at 48 h-intervals for 6 weeks induced disturbances in the neuroendocrine control of testosterone secretion, depletion in cauda epididymal sperm reserves without histopathological changes in the testis^11^.

The increase in intracellular calcium concentration^12^ and the disturbance in cellular redox potential secondary to activation of Krebs cycle^13^ are the major contributors to acceleration of programmed cell death by MSG. Its suppressive effect on hypothalamic–pituitary–gonadal axis adds another dimension by disrupting the hormonal commanding mechanisms. It has deteriorating effects on the oxidative status and histo-architecture of testis^7^ along with its ability to trigger apoptosis in the germ cells^1^.

Medicinal herbs contain a plenty of phytochemical antioxidants which gain popularity for their wide safety margin, outstanding efficacy and low price. Among them, *Nigella* *sativa* L. (NS) has the upper hand in defeating several health problems in Muslim countries and over the world^14^. Literature is punctuated with several evidence indicating the testicular protective properties of *Nigella* *sativa* L. seeds (NSS) in pesticides, chemotherapies and heavy metals challenged and diabetic rat models. NS oil mitigated the adverse effects induced by acetamiprid on the semen characteristics, testosterone and thiobarbituric acid-reactive substances^15^. NS oil in chlorpyrifos challenged rats boosted testosterone level, steroidogenic enzymes, semen quality and testicular morphological features^16^. Pre-treatment with NSS provided a scaffold against the disturbances in reproductive hormonal balance, semen outcomes and testicular histo-architecture in lead acetate intoxicant rat^17^. NS oil defeated cisplatin-induced testicular damage by suppressing lipid peroxidation and histological lesions^18^. Testosterone level in streptozotocin-induced diabetic rats restored without obvious change in luteinizing hormone level following supplementation with NSS powder^19^. NSS aqueous extract succeeded in improving testosterone level and redox potential biomarkers of the testis and accessory sexual glands in alloxan-induced diabetic rats^20^. NSS possesses amazing phytochemical profile that can combat feed additives-induced toxicities by suppressing free radical overproduction and enhancing redox circuitry^21^. For instance, thymoquinone, one of its most prominent phytochemical ingredients, acts as a highly potent testicular protectant through its antioxidant, anti-apoptotic and endocrine modulatory activities^22^. The aforementioned data provide a solid-based rationality to assume that dietary inclusion with NSS could be an effective candidate against MSG-induced testicular deteriorations. Lack of scientific articles in the literature exploring the potential protective effects of NSS on MSG-associated testicular dysfunctions adds fuels to our study to investigate this issue.

## Methods

### Identification of phytochemical constituents of *Nigellasativa* L. seeds

Chemical constituents of NSS were determined using gas chromatography/mass spectrophotometry (Triple Quadrupole) (GC-MS, 7890A-5975B, Agilent Technologies, USA), coupled with fused silica DB-5 capillary column (30 m length, 0.250 mm internal diameter, and 0.250 µm film thickness). A sample from the seeds was ground. One gram of the seed powder was weighted followed by addition of 1.5 ml of chloroform. The mixture was sonicated at 35 °C for one minute followed by centrifugation at 8000 rpm for 15 min at 40 °C. Then, the clear organic layer was withdrawn followed by injection in GC–MS. The column initially was held for 2 min at 40 °C, then increased to 150 °C at 10 °C/min, and held for 3 min, then raised to 220 °C with the same rate and held for 6 min, then raised to 280 °C and held for 15 min. 1 µl of the sample was injected with splitless injection mode. The carrier gas was helium with a flow rate of 0.5 ml/min for 10.9 min, then 1 ml/min for 30 min. The injector and detector temperature were 250 °C and 290 °C, respectively. Mass spectra were scanned in the range of 40–1000 amu. The scan time was 5 scans/s. The constituents were identified by the combination of retention index data and mass spectra using Wiley library. All studies of *Nigella* *sativa* L. seeds were carried out in accordance with the relevant institutional and international guidelines and regulations.

### Treatments

MSG powder was obtained in a sealed bottle from Morgan Chemical Industry, Egypt (purity 99%). NSS were obtained in a sealed bottle from Imtenan Health Shop Company, Obour City, Egypt.

### Animals and experimental design

A total of 18 healthy adult male albino rats aged 2–3 months (237 ± 32 g in weight) were used in this work. Animals were obtained from the Animal House of Faculty of Medicine, Assiut university, Assiut, Egypt. Rats were maintained in metal cages at room temperature with 12 h light: dark schedule during the period of the experiment. Water and food (standard rat chow) were allowed to rats ad libitum. The experimental protocol was approved by the Local Ethical Committee and by the Institutional Review Board of Faculty of Medicine, Assiut University (Approval Number: 17300469) and was carried out in accordance with relevant guidelines and regulations. This research was done in compliance with the ARRIVE guidelines and regulations (https://arriveguidelines.org). After 1 week of acclimatization, rats were randomly and equally divided into three groups. The control group received no treatment, the monosodium glutamate (MSG) group supplemented with MSG at a concentration of 30 g/kg feed^23^ thoroughly mixed with the ration for 21 days. MSG + NSS group administrated MSG at the same previous concentration together with NSS at a concentration of 30 g/kg feed^24,25^ for the same period.

### Sample collection

At the end of the experiment, venous blood samples were obtained immediately from the retro-orbital sinus of overnight fasted rats using heparinized microcapillary tubes. Blood samples were collected and centrifuged at 3000 rpm for 15 min to obtain serum which kept at − 20 °C until measurement of sexual hormones and oxidant/antioxidant parameters.

Rats were euthanized by cervical dislocation for tissue specimen collection. Orchidectomy was performed by open castration technique through a midline incision, and the testis was milked out of the incision site and rapidly exposed by incising the tunica vaginalis. The testis was fixed in 10% neutral buffered formalin, and then embedded in paraffin to be used in histopathological, histomorphometrical and immunohistochemical examination.

### Measurements of sexual hormones and oxidant/antioxidant profile

Testosterone level was estimated based on enzyme-linked immunosorbent assay (ELISA) by rat testosterone ELISA kit (catalog number: 80550) according to the manufacturer^'^s instruction (Crystal Chem, USA). The assay sensitivity is 0.066 ng/mL and the coefficient of variability is less than 10%.

Rat luteinizing hormone (LH) ELISA kit (catalog number: E-EL-R0026, Elabscience, USA) was used for measurement of LH level. The assay sensitivity is 0.94 mIU/mL and the intra- and inter-assay coefficient of variability are 4.33 and 5.36%, respectively. Lipid peroxides (LPO) were estimated according to a previous method^26^. Nitric oxide (NO) was measured based on an earlier procedure^27^. Superoxide dismutase (SOD) was estimated using a colorimetric kit (catalog number: SD2521, Biodiagnostic, Giza, Egypt) based on the ability of the enzyme to inhibit the phenazine methosulphate-mediated reduction of nitroblue tetrazolium dye. Glutathione-*S*-transferase (GST) was estimated by measuring the conjugation of 1-chloro-2,4-dinitrobenzene with reduced glutathione using the ultraviolet method^28^. Total antioxidant capacity (TAC) was assessed according to the manufacturer's instructions using commercial colorimetric kit (Catalog number: TA 2513) provided by Egyptian Company for Biotechnology, Cairo, Egypt.

### Histological examination

Transverse serial sections of 5 μm thickness were stained with the hematoxylin and eosin (H and E), Masson’s trichrome stain, Crossmon’s trichrome technique^29^, and Periodic Acid Schiff (PAS) reaction^30^ for neutral mucosubstances in seminiferous tubules (ST) and were examined by the light microscope. Negative image analysis was performed using CMEIAS (Center for Microbial Ecology Image Analysis System) color segmentation to assess the complex color micrographs that were obtained and to give more details^25,31,32^.

### Histomorphometric study

Image analysis system (Leica Quin 500 C Image analyzer computer system, Leica Imaging System LTD., Cambridge, England) at the department of Human Anatomy and Embryology, Faculty of Medicine, Assiut University, Egypt was used to measure:

1. The diameter of ST (μm) at six different sites in each H and E-stained section at a magnification of 200 and the average was calculated.
2. The number of cell layers of ST at six different sites in each H and E-stained section at a magnification of 200 and the average was calculated.
3. The height of the epithelium of ST (μm) at six different sites in each H and E-stained section at a magnification of 200 and the average was calculated.
4. The thickness of the basement membrane of ST (μm) at six different sites in each PAS-stained section at a magnification of 200 and the average was calculated.

### Immunohistochemistry of caspase-3, glutathione reductase (GR) and superoxide dismutase 2 (SOD2)

For the immunohistochemistry, we followed a previously published protocol^33^ sections of paraffin-embedded tissues were dewaxed, rehydrated, and rinsed in phosphate buffered saline (PBS). For antigen retrieval, the slides were placed in 10 mM sodium citrate buffer (pH 6.0) and heated to near boiling (95–98 °C) in a water bath for 20 min^34^ Endogenous peroxidase was inhibited by incubating the slides in 3% hydrogen peroxide for 10 min at room temperature before washing the slides in PBS (3 times for 5 min each)^33^. They were then processed for the different antibodies’ protocols described below^34–36^.

The sections were incubated overnight at 4 °C in a humid chamber with rabbit polyclonal antibody against caspase-3, marker for apoptosis^34^ (Abcam, Cambridge, Massachusetts, USA; 1:1000 dilution), or with rabbit polyclonal anti-GR and anti-SOD2, respectively (Chongqing Biospes Co., Ltd, China)^35,36^.

Thereafter, for the immunohistochemical detection of caspase-3, Avidin–Biotin-Peroxidase technique^33^ was used. The negative control sections were prepared without using the primary antibodies^33^. The positive control tissue was a specimen of human tonsil. Caspase-3 positive immunostained cells showed clear evident brown cytoplasmic coloration^34^.

For immunohistochemical detection of GR and SOD2, we used Power-Stain 1.0 Poly HRP DAB Kit for Mouse + Rabbit (Genemed Biotechnologies, South San Francisco, CA, USA)^35,36^.

All staining preparations were examined with an Olympus BX51 microscope, and photos were taken by an Olympus DP72 camera attached to the microscope^35^.

### Statistical analysis

GraphPad Prism Software version 5 (GraphPad Software Inc., La Jolla, CA, USA) was used for data analysis. Data were presented as mean ± standard error of the mean (SEM). Comparison among groups was carried out using One-Way Analysis of Variance (ANOVA) followed by Duncan posthoc test. Differences of *P* < 0.05 were considered to be statistically significant.

## Results

### Bioactive constituents of *Nigellasativa* L. seed

GC–MS of NSS revealed presence of 27 bioactive phytochemical constituents (Table 1). The major compounds were 9,12-octadecadienoic acid (88.40%), hexadecanoic acid (7.48%), and thymoquinone (1.5%).

**Table 1:** Chemical composition of *Nigella* *sativa* L. seed detected by gas chromatography/mass spectrophotometry.

Compounds	Retention time (min)	Area of component (%)	Molecular weight	Matching factor (%)
9,12-Octadecadienoic acid	23.430	88.400	294.256	99
Hexadecanoic acid	20.986	7.478	256.240	99
Thymoquinone	12.364	1.502	164.100	93
9,12-Octadecadienoic acid, methyl ester	21.918	0.795	294.256	99
9 Octadeceneoic acid, methyl ester	21.976	0.388	296.272	98
Hexadecanoic acid methyl ester	20.145	0.347	270.256	99
Farnesol isomer a	31.776	0.203	222.198	89
Ent-pimara-8(14),15-diene	20.501	0.176	272.250	93
2-Amino-3-cyano-4-(p,p′-diphenyl)-4-phenyl-5-(4′-methoxyphenyl)-3H-pyrrole	40.024	0.112	441.184	73
Longifolene	13.845	0.082	204.188	99
Hexanoic acid, 2-methyl-3-oxo-, ethyl ester	10.203	0.043	172.110	58
Tetradecanoic acid	18.871	0.042	228.209	93
2-Methoxy-4-ethyl-6-methylphenol	17.448	0.033	166.099	87
4-Tert-Butylpyrocatechol	17.448	0.033	166.099	86
(-)-Trans pinnae	19.110	0.030	138.141	69
Mixture of: 5,6-Dihydro-6-methyl-2H-pyran-2-one and 5-methoxy-3-pentene-2-ol	19.990	0.029	112.052	37
n-Nonadecane	19.809	0.022	268.313	98
2-[12-(2-Oxiranyl)dodecyl]oxirane	19.596	0.022	254.225	77
4-Methoxy-2,3,6-trimethylphenol	17.972	0.021	166.099	75
2-Bromo dodecane	14.654	0.018	248.114	91
3,4-Dimethyl-2-phenyl-, (2r-trans)-morpholine	17.448	0.033	191.131	88
Squalane	20.663	0.016	410.391	93
Citronellol	19.389	0.014	156.151	75
Cyclotetradecane	20.747	0.014	196.219	96
14.Alpha.-cheilanth-13(14)-enic methyl ester	14.175	0.010	386.318	69
N-(Trifluoroacetyl)-*N*,*O*,*O*′,*O*″-tetrakis(trimethylsilyl)norepinephrine	16.387	0.009	553.214	77
Trans-1-((2:2′,5′:2″-terthiophene)-3″-yl)-2-(4″″-cyanophenyl)ethene	13.638	0.008	375.021	95

### Effects of *Nigella sativa* L. seeds on sexual hormones and oxidant/antioxidant profile of monosodium glutamate challenged rats

As shown in Table 2, disruption of pituitary–gonadal axis in MSG challenged rats was manifested by a significant decrease in testosterone level along with a significant increase in LH level, whereas NSS restored the sexual hormonal balance towards the normal level. The exposure to MSG was responsible for induction of oxidative stress status evident by a significant increase in LPO, GST and TAC and a significant decrease in NO and SOD. Dietary supplementation of NSS to MSG intoxicated rats succeeded in rebalance some aspects of oxidant/antioxidant profile such as LPO and TAC without causing any significant change in NO, SOD and GST when compared with the MSG group.

**Table 2:** Effect of *Nigella* *sativa* L. seed on serum luteinizing hormone, testosterone and oxidative stress parameters of rats suffer from monosodium glutamate-induced testicular dysfunction.

Group	Control	MSG	MSG + NSS	P value
**Parameter**				
LH level (mU/ml)	16.167 ± 2.218^b^	37.733 ± 2.270^a^	21.800 ± 2.590^b^	0.002
Testosterone level (ng/ml)	2.600 ± 0.116^a^	1.650 ± 0.144^b^	2.400 ± 0.252^a^	0.022
LPO level (nmol/mL)	1.161 ± 0.139^b^	2.260 ± 0.130^a^	1.079 ± 0.165^b^	0.000
NO level (nmol/mL)	99.603 ± 5.541^a^	52.565 ± 4.769^b^	70.308 ± 7.097^b^	0.001
SOD activity (U/mL)	73.643 ± 2.081^a^	62.984 ± 2.340^b^	58.462 ± 4.130^b^	0.011
GST activity (U/L)	16.633 ± 1.637^b^	37.020 ± 3.298^a^	30.750 ± 3.031^a^	0.005
TAC (mM/L)	0.544 ± 0.046^b^	0.996 ± 0.135^a^	0.620 ± 0.038^b^	0.017

*MSG* monosodium glutamate, *NSS* *Nigella* *sativa* L. seed, *LH* luteinizing hormone, *LPO* lipid peroxides, *NO* nitric oxide, *SOD* superoxide dismutase, *GST* glutathione-*S*-transferase, *TAC* total antioxidant capacity.

Results are expressed as the mean ± SEM of 6 rats per group.

^a,b^Different letters indicate significant differences at *P* < 0.05 (one-way ANOVA followed by Duncan’s posthoc-test).

### Effects of *Nigella sativa* L. seeds on the histopathological features of testis of monosodium glutamate challenged rats

**Figure 1:**
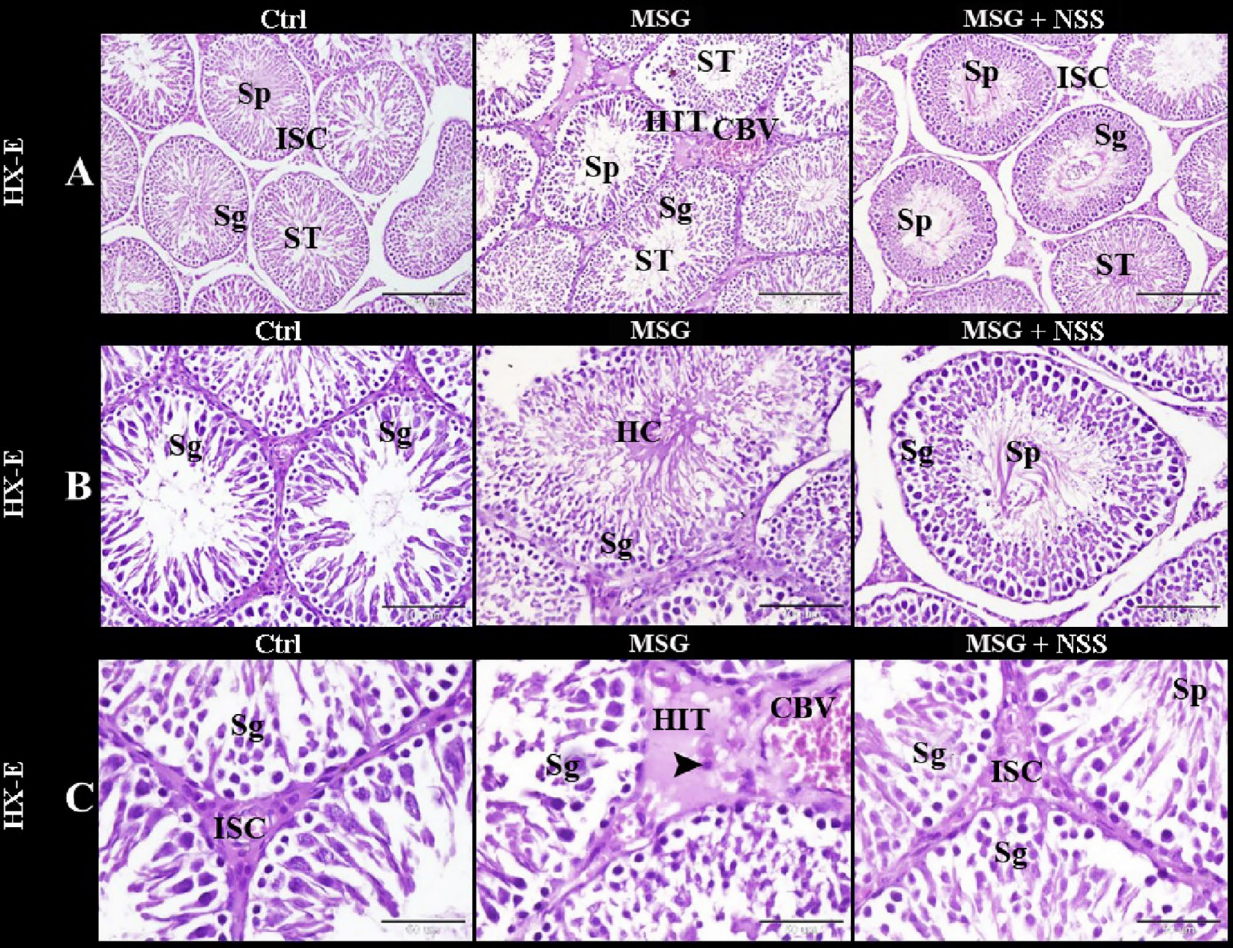
Photomicrograph of paraffin sections in the rats' testes showed the protective effect of NSS on MSG induced testicular damages. Control group (Ctrl) in (**A**–**C**) showed the normal architecture of the testis which formed of regular seminiferous tubules (ST) separated by numerous interstitial cells of Leydig (ISC). The seminiferous tubules were lined by stratified germinal epithelium which represents the spermatogenic cells (Sg) in different stages of development up to mature sperm. The seminiferous tubules had narrow lumen filled with mature sperms (Sp). MSG group in (**A**–**C**) showed irregular seminiferous tubules (ST) separated by hyalinized interstitial tissue (HIT) with apoptotic interstitial cells (arrowhead) and congested blood vessels (CBV). The seminiferous tubules were lined by few layers of the spermatogenic cells (Sg). The seminiferous tubules had a wide lumen with hyalinized center (HC) or contained few numbers of sperms (Sp). MSG + NSS group in (**A**–**C**) showed that the architecture of the testis was retained to normal. It was formed of regular seminiferous tubules (ST) separated by numerous interstitial cells of Leydig (ISC). The seminiferous tubules were lined by stratified germinal epithelium which represents the spermatogenic cells (Sg) in different stages of development up to mature sperm. The seminiferous tubules had narrow lumen filled with mature sperms (Sp). Original magnification; (**A**) ×100, scale bar 200 μm; (**B**) ×200, scale bar 100 μm; (**C**) ×400, scale bar 50 μm, Hematoxylin and Eosin stain.

**Figure 2:**
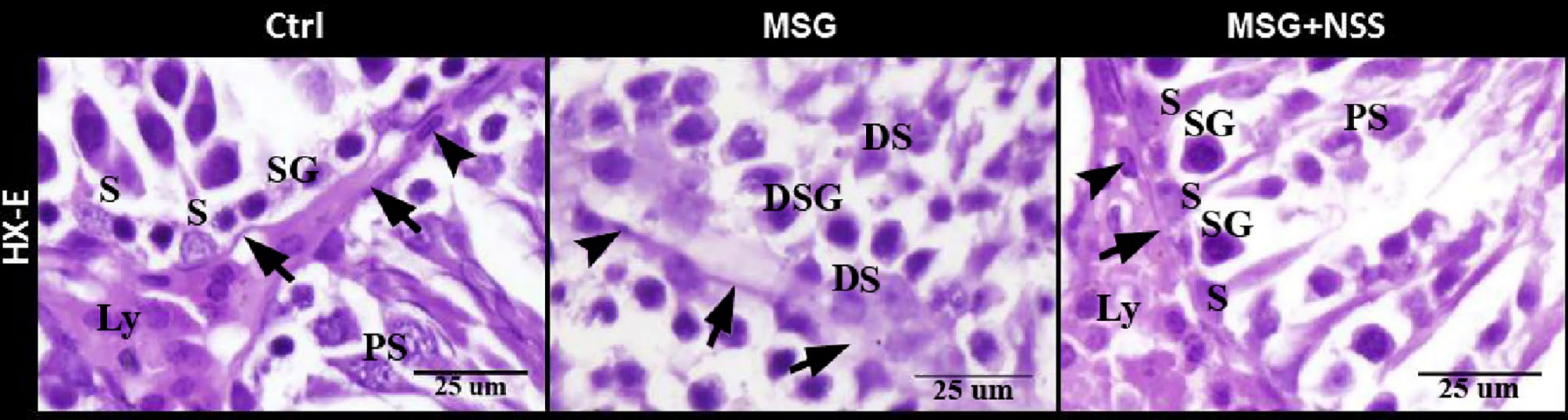
Photomicrograph of paraffin sections in the rats' testes showed the protective effect of NSS on MSG induced testicular damages. Control group (Ctrl) showed the normal healthy Sertoli cells (S) adhered to the continuous basement membrane (arrow), spermatogonia (SG) in the basal compartment next to the basement membrane, the large primary spermatocytes (PS) with filamentous chromosomes next to spermatogonia, flattened smooth muscle like myoid cells (arrow head) outside the basement membrane and Leydig cells (Ly) which were large polygonal cells with rounded centrally located nuclei and vacuolated acidophilic cytoplasm. MSG group showed degenerated Sertoli cells (DS) away from the disrupted basement membrane (arrows), degenerated spermatogonia (DSG) away from the basal compartment, degenerated primary spermatocytes (DS), degenerated myoid cells (arrowhead). MSG + NSS group showed the normal healthy Sertoli cells (S) adhered to the continuous basement membrane (arrow), spermatogonia (SG) in the basal compartment next to the basement membrane, the large primary spermatocytes (PS) next to spermatogonia, flattened smooth muscle like myoid cells (arrow head) outside the basement membrane and Leydig cells (Ly) which were large polygonal cells with rounded centrally located nuclei and vacuolated acidophilic cytoplasm. Original magnification; ×400, scale bar 25 μm, Hematoxylin and Eosin stain.

The histological examination of the testes in the control group showed normal architecture of the testis which formed of regular ST separated by numerous interstitial cells of Leydig. ST were lined by stratified germinal epithelium (3–7 layers) and Sertoli cells. The stratified germinal epithelium was represented by the spermatogenic cells in different stages of development up to mature sperm. These cells were spermatogonia in the basal compartment next to the basement membrane, the large primary spermatocytes with filamentous chromosomes next to spermatogonia, spermatid with deeply stained rounded nucleus, and the mature sperms. ST had narrow lumen filled with mature sperms (Fig. 1: Ctrl; A–C). The normal healthy Sertoli cells were tall pyramidal cells adhered to the continuous basement membrane and had large oval basally located nuclei. The flattened smooth muscle like myoid cells outside the basement membrane were observed. Leydig cells were large polygonal cells with rounded centrally located nuclei and vacuolated acidophilic cytoplasm (Fig. 2: Ctrl).

In MSG group, the testes showed irregular ST separated by hyalinized interstitial tissue with apoptotic interstitial cells and congested blood vessels. ST were lined by few layers of the spermatogenic cells. It had a wide lumen with hyalinized center or contained few numbers of sperms (Fig. 1: MSG; A–C). MSG group also showed degenerated Sertoli cells away from the disrupted basement membrane, degenerated spermatogonia away from the basal compartment, and degenerated primary spermatocytes and myoid cells (Fig. 2: MSG).

In MSG + NSS group, the architecture of the testis was returned to the normal. It was formed of regular ST separated by numerous interstitial cells of Leydig. ST were lined by stratified germinal epithelium which represents the spermatogenic cells in different stages of development up to mature sperm. It had narrow lumen filled with mature sperms (Fig. 1: MSG + NSS; A–C). MSG + NSS group also showed the normal healthy Sertoli cells adhered to the continuous basement membrane, spermatogonia in the basal compartment next to the basement membrane, the large primary spermatocytes next to spermatogonia, flattened smooth muscle like myoid cells outside the basement membrane, and Leydig cells which were large polygonal cells with rounded centrally located nuclei and vacuolated acidophilic cytoplasm (Fig. 2: MSG + NSS).

**Figure 3:**
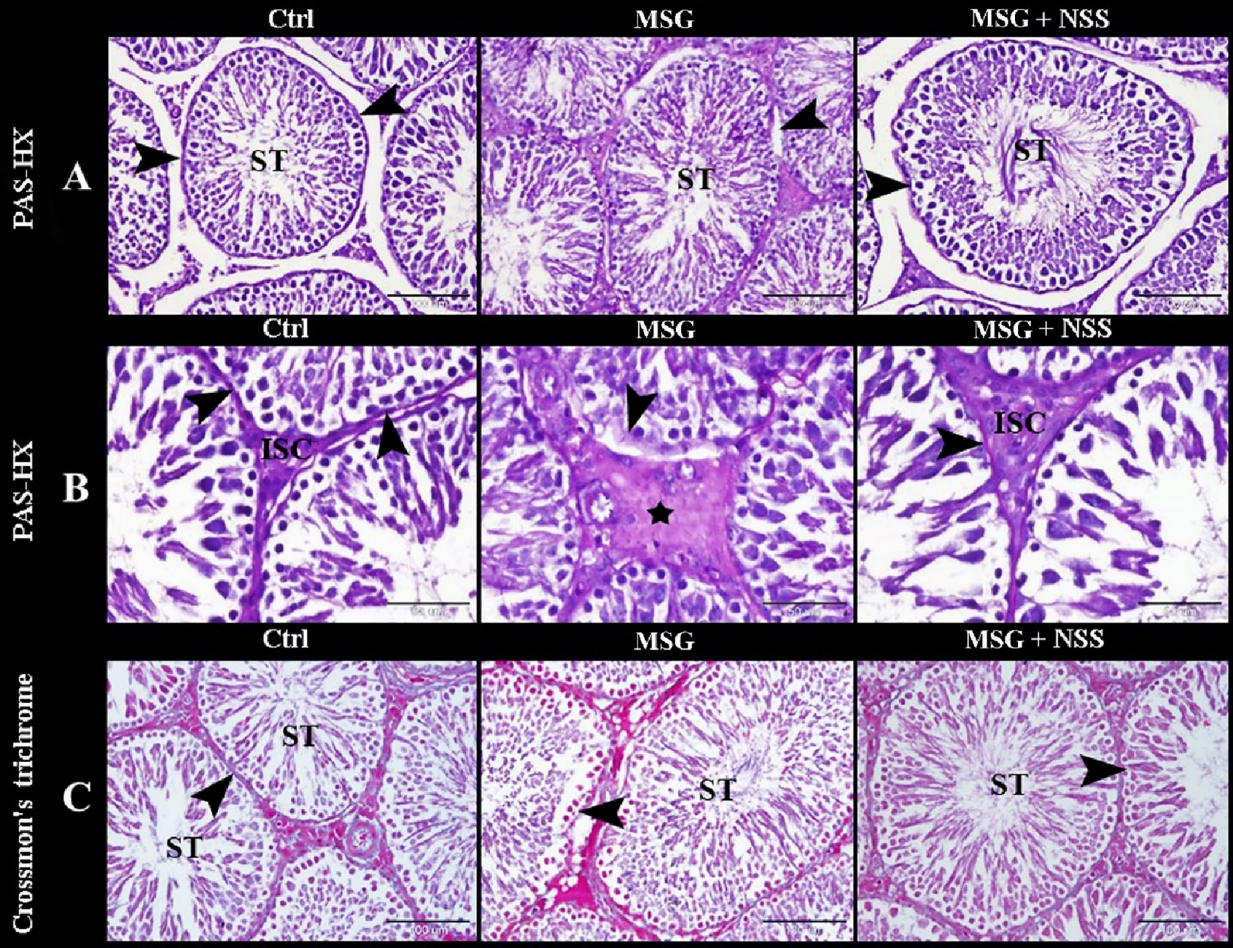
Photomicrograph of paraffin sections in the rats' testes showed the protective effect of NSS on MSG induced testicular damages. Control group (Ctrl) in (**A**,**B**) showed the seminiferous tubules (ST) with regular continued PAS positive basement membrane (arrowheads) separated by numerous interstitial cells of Leydig (ISC). MSG group in (**A**,**B**) showed the seminiferous tubules (ST) with irregular interrupted PAS positive basement membrane (arrowhead) separated by hyalinized interstitial tissue (star) and congested blood vessels (CBV). MSG + NSS group in (**A**,**B**) showed the seminiferous tubules (ST) with regular continued PAS positive basement membrane (arrowhead) separated by numerous interstitial cells of Leydig (ISC). Control group (Ctrl) in (**C**) showed the seminiferous tubules (ST) with normal peritubular collagen fibers (arrowhead). MSG group in (**C**) showed the seminiferous tubules (ST) with few, irregular and interrupted peritubular collagen fibers (arrowhead). MSG + NSS group in (**C**) showed the seminiferous tubules (ST) with regular continued peritubular collagen fibers (arrowhead). Original magnification; (**A**) ×200, scale bar 100 μm, periodic acid-Schiff (PAS) and hematoxylin, (**B**) ×400, scale bar 50 μm, periodic acid-Schiff (PAS) and hematoxylin; (**C**) ×200, scale bar 100 μm, Crossmon’s trichrome technique.

**Figure 4:**
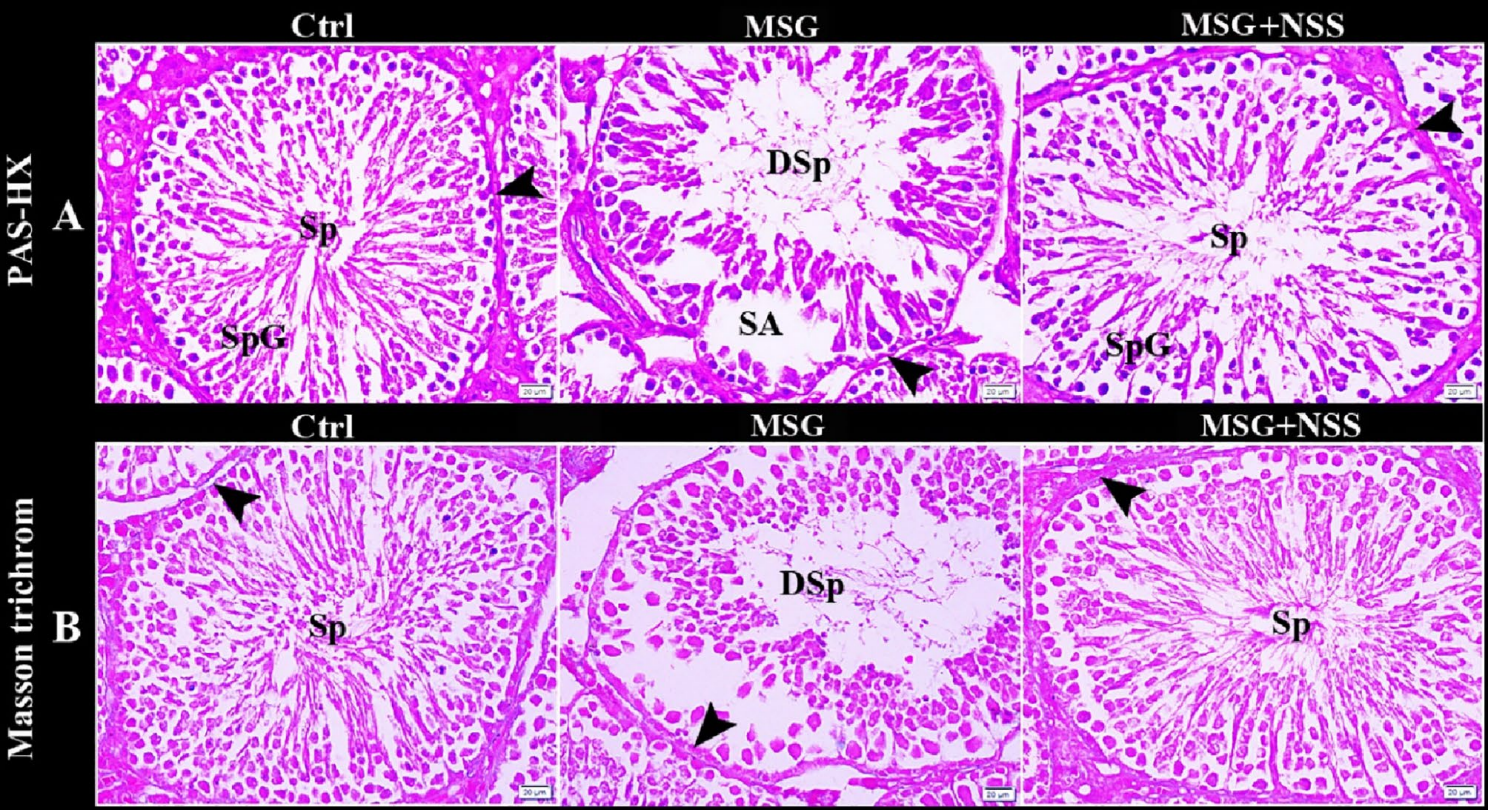
Photomicrograph of paraffin sections in the rats' testes showed the protective effect of NSS on MSG induced testicular damages. Control group (Ctrl) in (**A**) showed the seminiferous tubules with regular continued PAS positive basement membrane (arrowheads). Note the normal healthy developing spermatogenic cells (SpG) and the healthy sperms (Sp) in the lumen of the seminiferous tubules. MSG group in (**A**) showed the seminiferous tubules with irregular interrupted PAS positive basement membrane (arrowhead). Note the spermatogenic arrest (SA) and the degenerated sperms (DSp) in the lumen of the seminiferous tubules. MSG + NSS group in (**A**) showed the seminiferous tubules (ST) with regular continued PAS positive basement membrane (arrowhead). Note the normal healthy developing spermatogenic cells (SpG) and the healthy sperms (Sp) in the lumen of the seminiferous tubules. Control group (Ctrl) in (**B**) showed the seminiferous tubules with normal peritubular collagen fibers (arrowhead) and the healthy sperms (Sp) in the lumen of the seminiferous tubules. MSG group in (**B**) showed the seminiferous tubules with few, irregular and interrupted peritubular collagen fibers (arrowhead) and the degenerated sperms (DSp) in the lumen of the seminiferous tubules. MSG + NSS group in (**B**) showed the seminiferous tubules with regular continued peritubular collagen fibers (arrowhead) and the healthy sperms (Sp) in the lumen of the seminiferous tubules. Original magnification; (**A**) ×200, scale bar 20 μm, periodic acid-Schiff (PAS) and hematoxylin, (**B**) ×200, scale bar 20 μm, Masson’s trichrome technique.

**Figure 5:**
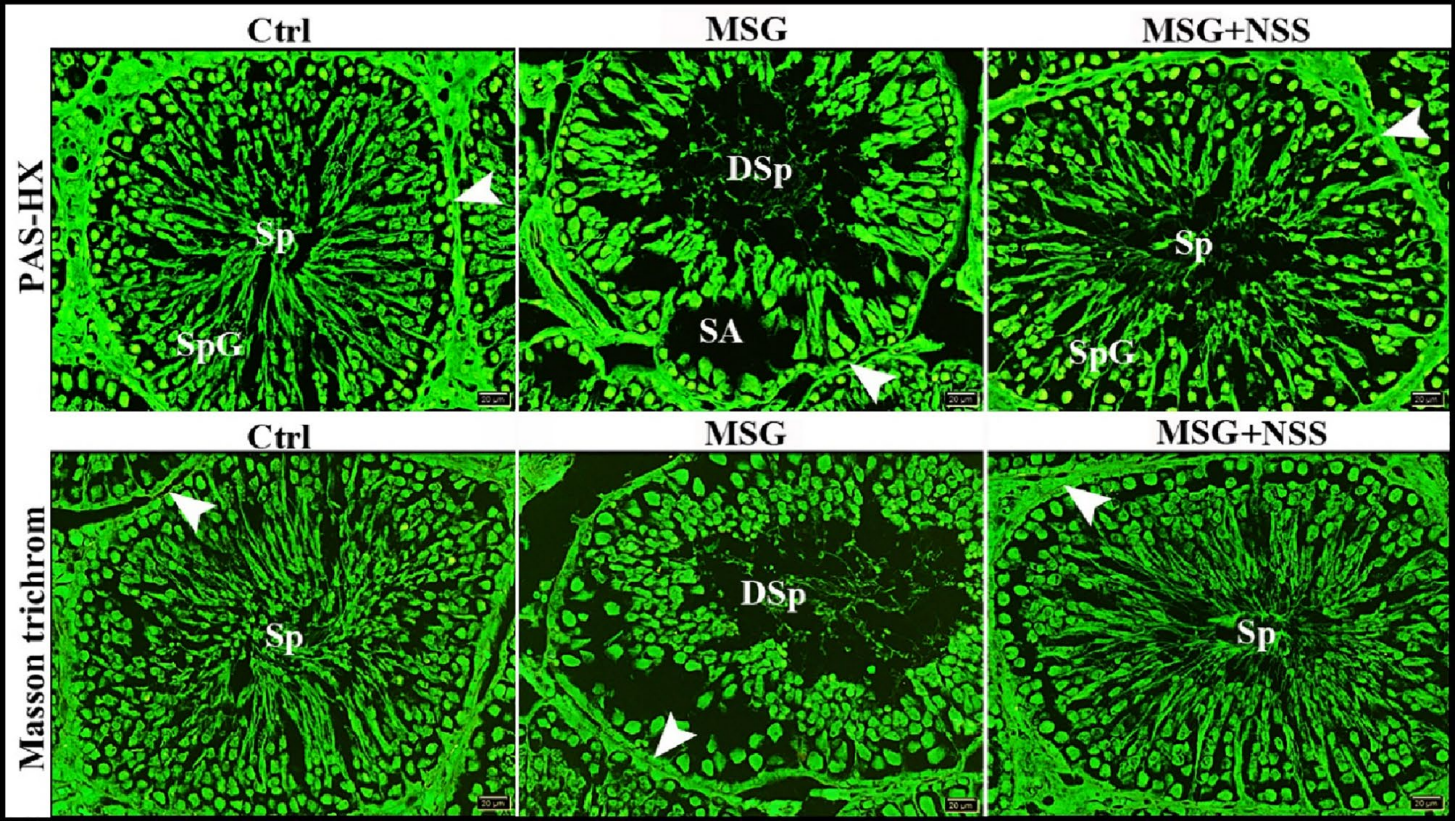
Negative images of the photomicrographs shown in Fig. 4 were analyzed using CMEIAS Color Segmentation 1.0 Software to assess the complex color micrographs and to give more details. https://mybiosoftware.com/cmeias-color-segmentation-1-0-segment-analyze-foreground-objects-complex-images.html.

We used PAS technique to evaluate the regularity and integrity of basement membranes of ST. The control group showed ST with regular continued PAS-positive basement membranes. While MSG group showed ST with irregular interrupted PAS-positive basement membranes. Whereas MSG + NSS group showed ST with regular continued PAS-positive basement membranes (Figs. 3A,B, 4A). To evaluate the distribution and arrangement of the peritubular collagen fibers, we used Crossmon’s and Masson’s trichrome techniques. The control group showed ST with normal peritubular collagen fibers. While MSG group showed ST with few, irregular and interrupted peritubular collagen fibers. Whereas MSG + NSS group showed ST with regular continued peritubular collagen fibers (Figs. 3C, B). We observed that there were spermatogenic arrest and degenerated sperms in MSG group compared to the normal healthy developing spermatogenic cells and the healthy sperms in MSG + NSS group which resembled the control group (Fig. 4A). Figure 5 shows negative images of the photomicrographs shown in Fig. 4 give more clear evidence about the process of spermatogenesis and evaluate the regularity and integrity of basement membranes of ST.

### Effects of *Nigella sativa* L. seeds on the testicular morphometric indices of monosodium glutamate challenged rats

**Figure 6:**
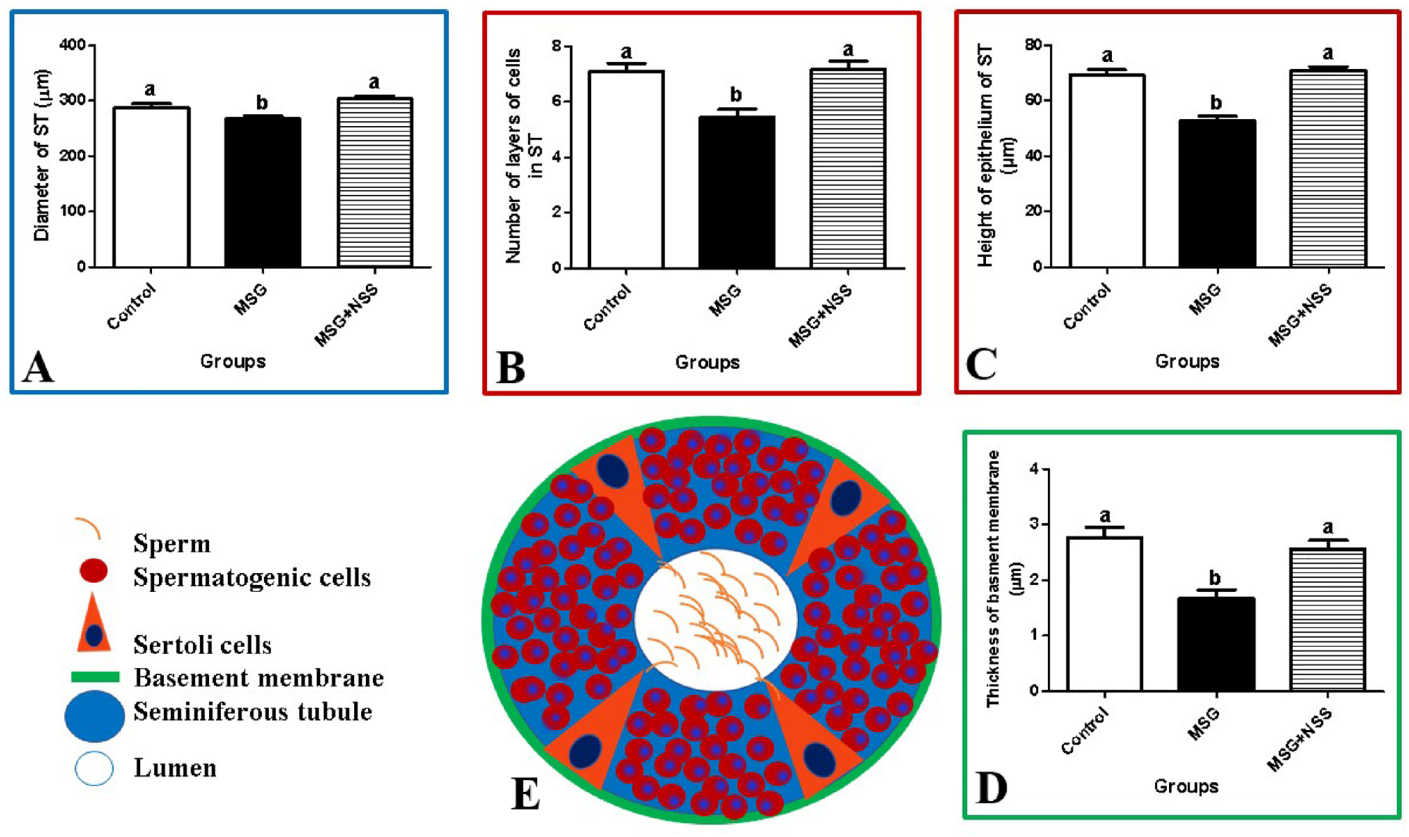
Showed the morphometrical results obtained in the current study analyzed using GraphPad Prism Software version 5 (GraphPad Software Inc., La Jolla, CA, USA) https://www.graphpad.com/scientific-software/prism/. (**A**) Showed that MSG significantly decreased the diameter of the seminiferous tubules and the addition of NSS kept the normal diameter of the seminiferous tubules. (**B**) Showed that MSG significantly decreased the number of spermatogenic cell layers which lined the seminiferous tubules and the addition of NSS kept the normal number of spermatogenic cell layers. (**C**) Showed that MSG significantly decreased the height of the epithelium of the seminiferous tubules and the addition of NSS kept the normal height of the epithelium of the seminiferous tubules. (**D**) Showed that MSG significantly decreased the thickness of the basement membrane of the seminiferous tubules and the addition of NSS kept the normal thickness of the basement membrane of seminiferous tubules. (**E**) Showed a diagram drawn by the author using Microsoft PowerPoint 2010 program to illustrate the general histological structure of the seminiferous tubules. https://www.microsoft.com/en-eg/download/details.aspx?id=20873.

The histomorphometric analysis in MSG group indicated tubular atrophy evident by a significant reduction in thickness of basement membrane, number of cellular layers in ST, diameter of ST and height of their lining epithelium (Table 3; Fig. 6). On the other hand, all studied histomorphometric outcomes were normalized in MSG + NSS group.

**Table 3:** Effect of *Nigella* *sativa* L. seed on some testicular morphometrical parameters of rats suffer from monosodium glutamate-induced testicular dysfunction.

Group	Control	MSG	MSG + NSS	P value
**Parameter**				
Thickness of basement membrane (µm)	2.773 ± 0.182^a^	1.670 ± 0.151^b^	2.556 ± 0.154^a^	0.000
Diameter of ST (µm)	286.860 ± 7.865^a^	267.520 ± 4.987^b^	303.300 ± 5.620^a^	0.002
Number of cell layers of ST	7.090 ± 0.315^a^	5.450 ± 0.282^b^	7.180 ± 0.296^a^	0.000
Height of epithelium of ST (µm)	69.298 ± 1.912^a^	52.620 ± 1.856^b^	71.028 ± 1.328^a^	0.000

Results are expressed as the mean ± SEM of 6 rats per group.

*MSG* monosodium glutamate, *NSS* *Nigella* *sativa* L. seed, *ST* seminiferous tubules.

^a,b^Different letters indicate significant differences at *P* < 0.05 (one-way ANOVA followed by Duncan’s posthoc-test).

### Effects of *Nigella sativa* L. seeds on immuno-expression of caspase-3, superoxide dismutase 2 and glutathione reductase in the tests of monosodium glutamate challenged rats

**Figure 7:**
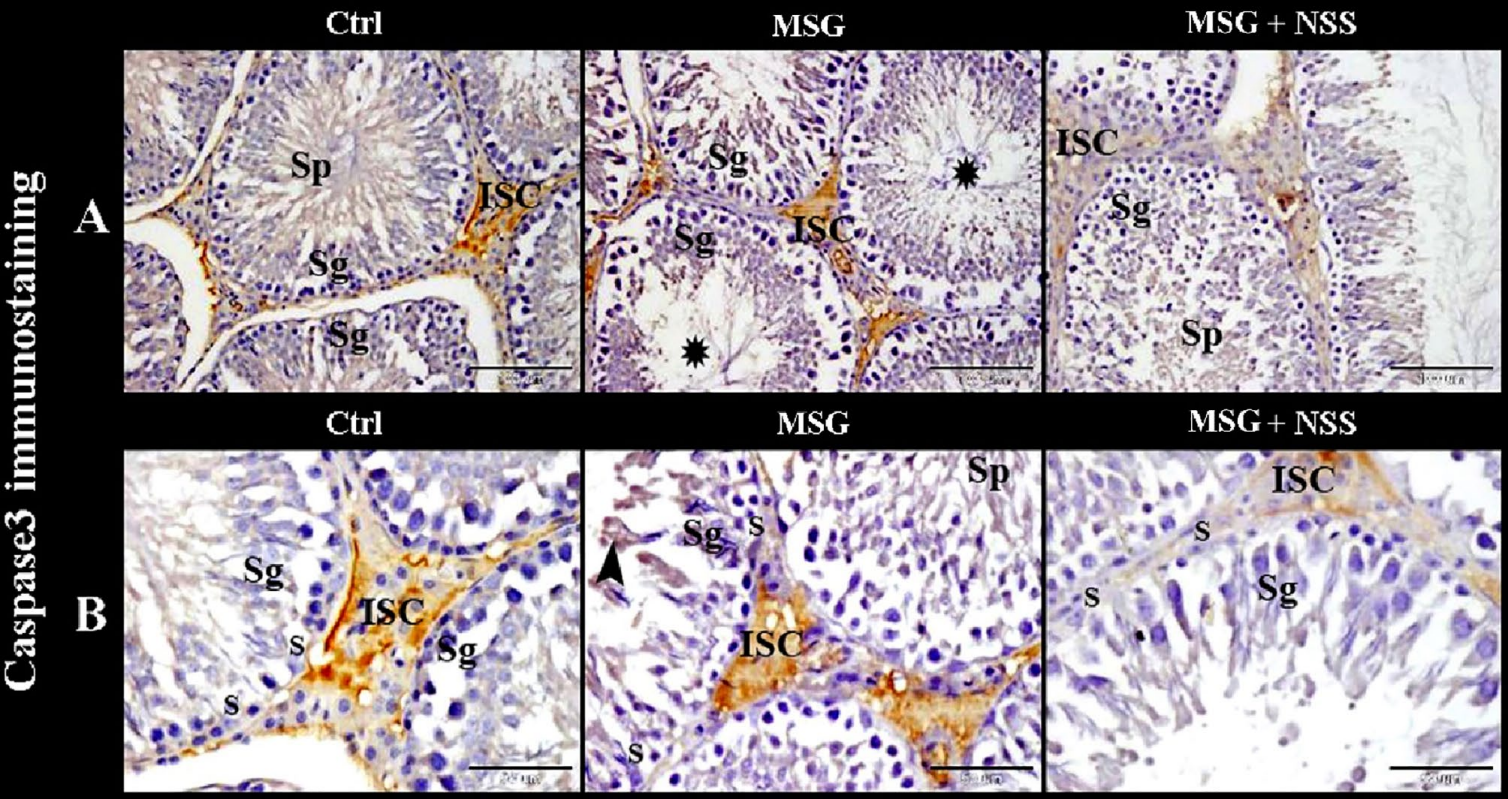
Photomicrograph of caspase-3 immunostaining in the rats' testes showed the protective effect of NSS on MSG induced testicular damages. Control group (Ctrl) in (**A**,**B**) showed negative to weak caspase-3 immunostaining in the spermatogenic cells (Sg), sperms (Sp), Sertoli cells (S) and interstitial cells of Leydig (ISC). MSG group in (**A**,**B**) showed significantly increased caspase-3 immunoexpression in the spermatogenic cells (Sg), spermatids (arrowhead), sperms (Sp), Sertoli cells (S) and interstitial cells of Leydig (ISC). Note the wide empty lumen (balck star) of some seminiferous tubules. MSG + NSS group in (**A**,**B**) showed negative to weak caspase-3 immunostaining in the spermatogenic cells (Sg), sperms (Sp), Sertoli cells (S) and interstitial cells of Leydig (ISC). Positive caspase-3 immunoreactivity presented as a brownish yellow color. Original magnification; (**A**) ×200, scale bar 100 μm; (**B**) ×400, scale bar 50 μm.

To evaluate the programmed cell death in the testes, we used caspase-3 immuno-expression. The control group showed negative to weak caspase-3 immunostaining in the spermatogenic cells, sperms, and Sertoli and Leydig cells. While MSG group showed a marked increase in caspase-3 immuno-expression in the spermatogenic cells, spermatids, sperms, Sertoli cells and Leydig cells. Whereas MSG + NSS group showed negative to weak caspase-3 immunostaining in the spermatogenic cells, sperms, and Sertoli and Leydig cells. Positive caspase-3 immuno-reactivity presented as a brownish yellow colour in the cytoplasm and /or nucleus of the cells (Fig. 7A,B).

**Figure 8:**
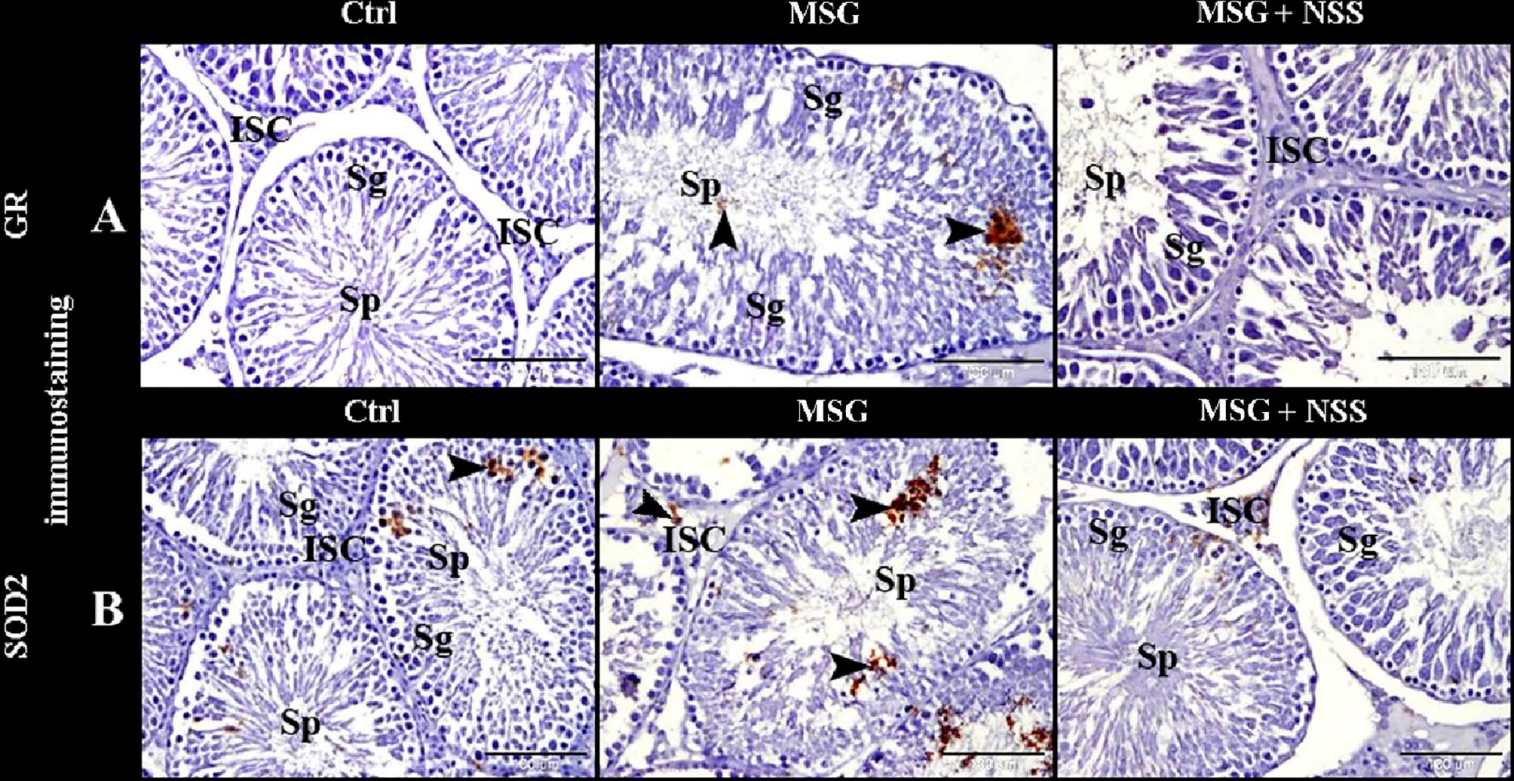
Photomicrograph of GR (**A**) and SOD2 (**B**) immunostaining in the rats' testes showed the protective effect of NSS on MSG induced testicular damages. (**A**) Control group (Ctrl) showed negative GR immunostaining in the spermatogenic cells (Sg), sperms (Sp) and interstitial cells of Leydig (ISC). MSG group showed positive GR immunoexpression (arrowheads) in the spermatogenic cells (Sg) and the sperms (Sp). MSG + NSS group showed negative GR immunostaining in the spermatogenic cells (Sg), sperms (Sp) and interstitial cells of Leydig (ISC). (**B**) Control group (Ctrl) showed few SOD2 immunostaining (arrowheads) in the spermatogenic cells (Sg) negative immunostaining in the sperms (Sp) and interstitial cells of Leydig (ISC). MSG group showed significantly increased SOD2 immunoexpression (arrowheads) in the spermatogenic cells (Sg) and interstitial cells of Leydig (ISC) and negative immunostaining in sperms (Sp). MSG + NSS group showed weak SOD2 immunostaining in the spermatogenic cells (Sg) and interstitial cells of Leydig (ISC) and negative immunostaining in the sperms (Sp). Positive GR or SOD2 immunoreactivity presented as a brownish color (arrowheads). Original magnification; (**A**,**B**) ×200, scale bar 100 μm.

To evaluate the oxidative damage in the different cells of the testes, we used GR and SOD2 immunohistochemistry. The control group showed negative GR immunostaining in the spermatogenic cells, sperms and Leydig cells (Fig. 8A, Ctrl). The control group also showed few SOD2 immunostaining in the spermatogenic cells, and negative immunostaining in the sperms and Leydig cells (Fig. 8B, Ctrl). While MSG group showed positive GR immuno-expression in the spermatogenic cells and sperms (Fig. 8A. MSG). MSG group also showed a significant increase in SOD2 immuno-expression in the spermatogenic and Leydig cells, and negative immunostaining in the sperms (Fig. 8B, MSG). Whereas MSG + NSS group showed negative GR immunostaining in the spermatogenic cells, sperms and Leydig cells (Fig. 8A. MSG + NSS). MSG + NSS group also showed weak SOD2 immunostaining in the spermatogenic and Leydig cells, and negative immunostaining in the sperms (Fig. 8B. MSG + NSS). Positive GR or SOD2 immuno-reactivity presented as a brownish colour in the cytoplasm and /or nucleus of the cells.

## Discussion

MSG consumption was incriminated in a wide array of health hazards including reproductive toxicity. The searching for phytochemical strategies having an effective protective role and a broad safety profile is worthwhile. In the current investigation, MSG in male rats was capable of triggering sexual hormonal and testicular redox imbalance and apoptotic cascade. Thus, it is important to reconsider the usage of MSG as a flavor enhancer. On the other side, NSS dietary intervention successfully mitigated the above-mentioned disorders via its antioxidant and cytoprotective properties. These findings are of major importance in presenting NSS as a potent testicular protectant in the field of herbal remedy especially in the light of wide spread utilization of food preservatives and high cost of bioactive food derivatives in the food industry.

The phytochemical characterization of NSS revealed presence of some bioactive constituents similar to that observed by^37^ providing explanation to its observed antioxidant and anti-apoptotic effects in this study. The fatty acid methyl esters in NSS are potent free radical scavengers, antioxidants and anticancer agents^38^. Squalene behaves as antioxidant by stimulating the total and phosphorylated nuclear factor E2-related factor 2 which in turn activates the transcription of antioxidant and cytoprotective enzymes^39^. Farnesol counteracts intrinsic apoptotic cascade through its antioxidant activity^40^. Thymoquinone provides a protective effect against reproductive dysfunction by downregulating testicular inducible nitric oxide synthase and nuclear factor kappa-B and upregulating aromatase protein expression^41^. However, there is a wide variety in the concentrations of phytochemical ingredients in NSS according to type of extraction. Both chloroform and petroleum ether extracts of NSS have antioxidant, free radical scavenging, and anti-inflammatory properties in stroke rat model^42^. The ethanol extract of NSS relieved nickel chloride-induced hepato-renal injury in rats by the attenuation of lipid peroxidation and enhancement of both catalase and GST activities^43^. Presence of polyphenol, flavonoids and volatile compounds in the ethanol extract is involved in its antioxidant, anti-inflammatory and anti-apoptotic activities^43,44^. The highest concentration of total phenolic compounds and flavonoids were found in the NS capsules and oil followed by the methanol and aqueous extracts, with a linear relationship between free radical inhibition and total polyphenols^45^. NSS oil has a high ratio of unsaturated per saturated fatty acids and the oil extracted with dichloromethane has the highest amounts of thymoquinone^46^. High level of antioxidants could be derived from NSS oil extracted by supercritical fluid extraction than cold press^47^. NSS oil possesses anti-inflammatory, antioxidant, immunomodulatory and anticancer activities^48^.

MSG represented a real challenge to the sexual hormonal balance by disrupting the regulatory mechanisms of pituitary–gonadal axis as confirmed by the findings of this study and others^11,38^. The diminution in hormonal secretory capacity of Leydig cells could be attributed to peroxidative damage, reduced count, and morphological and ultrastructural abnormalities^49^. One explanation for the reduction in testosterone output may be increased apoptotic cell death in Leydig cells as shown by the histopathological examination and immunohistochemistry technique in our study. Although apoptosis is an essential prerequisite in maintaining the population of Leydig cells and testosterone levels^50^ increased apoptosis leads to decline in testosterone production, which may increase germ cell apoptosis and the possibility of infertility^51^. However, other studies showed insignificant changes in testosterone levels following MSG challenge^7,52^. This differential response may be due to difference in the experimental design including the dose and duration of exposure. The raise in LH level of MSG group is secondary to the drop in testosterone secretion, as anterior pituitary gonadotrophs release from the negative feedback effect exerted by testosterone^53^.

The restoration of functional integrity of pituitary–gonadal axis by dietary inclusion of NSS in MSG challenged rats is similar to that found in mice exposed to carbendazim^54^. This outcome may be attributed to the ability of NSS to increase the testicular steroidogenic enzymes activities^55^. Thymoquinone diminishes the apoptotic changes in Leydig cells as reported in previous investigation^22^. NSS is a rich source of unsaturated fatty acids^46^ which stimulate the activity of 17 β-hydroxysteroid dehydrogenase^56^, a key regulatory enzyme in the pathway of testosterone biosynthesis, by changing phospholipid composition in the plasma membranes of testes which alters the receptor-mediated gonadotropin actions^57^. Conjugated linolenic acid in NSS, as octadecadienoic in Leydig tumour rat cells and trained mice, up-regulated specific genes encoding enzymes and transport proteins involved in testosterone biosynthesis^58^. Thymoquinone induced the transcript level of aromatase in the testis of rats exposed to lead^22^.

Previous studies support our findings of increased LPO level in MSG group as compared to the control one^38^. MSG activates xanthine oxidase, a superoxide-initiating enzyme, which produces a burst of oxygen free radicals eliciting lipid peroxidation through chain reactions^59^. Excessive amount of glutamate dissociated from exogenously supplemented MSG results in overproduction of free radicals by intracellular depletion of glutathione^60^ and elevation of oxidative phosphorylation and mitochondrial hyperpolarization^61^. It is well known that the attack of reactive oxidants to biological macromolecules, namely lipid, protein and DNA, is incriminated in the etiology and progression of testicular dysfunction.

The reduction in NO bioavailability in our MSG-induced testicular intoxicated rat model is compatible with that observed in the mesenteric arterioles of rats impairing its microvascular reactivity due to down-regulation of endothelial nitric oxide expression^62^. NO acts as a potent antioxidant-inducible mediator because it breaks down the free radicals-driven lipid peroxidative reactions^63^ enhances the antioxidant potency of reduced glutathione^64^ and up-regulates the gene expression of enzymatic antioxidants^65^. Therefore, decreased NO level in the serum of MSG group indicates attenuation in the cellular resistance in the face of oxidative and nitrosative stress. Taken into account that NO is strong vasodilator agent, its reduction can result in impairment in testicular morphology^66^ and hormonal androgenesis^67^ by compromising the microvascular blood flow. NO participates in stimulating Leydig cells to secrete testosterone^68^, and in controlling sperm capacitation and acrosomal reaction which are indispensable for sperm fertilizing capacity^69^. Accordingly, the reduction in NO level could be implicated in disrupting testicular steroidogenic biosynthesis and inducing infertility.

The reduction in NO level in the present study was observed in concomitant with the reduction in SOD activity suggesting a causal relationship. Given that SOD catalyzes the dismutation of superoxide radicals to molecular oxygen and hydrogen peroxide, the suppression in its activity tends to accumulate superoxide radicals which in turn interact with NO to limit its bioavailability^70^. In fact, NO is necessary for up-regulation of SOD expression to prevent superoxide radicals-mediated degradation of NO^71^.

Increased GST activity in MSG group indicates stimulation of detoxification process, as a part of body defensive mechanism, via catalyzing the conjugation of reduced glutathione to endogenous and exogenous toxic substances^72^ rendering them less harmful and enabling their elimination^73^.

The different response patterns of measured enzymatic antioxidants in the present study could be owing to variation in their inducibility at both transcriptional and translational levels^74^. In this prospective, TAC is regarded to be one of the most appropriate measures for estimating oxidative/reductive potency taking into consideration the cumulative synergistic action of overall antioxidants presents in the sample^75^. In contrast, the estimation of individual antioxidants may give a confusing picture because antioxidants perform their actions through chain-breaking reactions^76^. In accordance with earlier studies^52^, serum TAC was elevated representing a compensatory adaptive response to oxidative instability following MSG supplementation. Accumulated body of evidence denotes activation of a wide range of enzymatic and non-enzymatic antioxidants under MSG stress^77,78^. As stimulation of the overall antioxidant capacity was necessary to combat the detrimental effects of oxidative injury, the shift in redox balance towards the pro-oxidant side evokes up-regulation of endogenous antioxidant defenses mediated by activation of redox-sensitive transcription factors and its down-stream signaling pathways^79^. According to our findings, the rise in the antioxidant potency of the body is supported by the positive GR immuno-expression in the germ cells and up-regulation in SOD2 immuno-expression in the spermatogenic and Leydig cells.

The antioxidant effect of NS plays a pivotal role in protecting against the undesirable effects of feed additives by improving glutathione redox cycle^80^ and blocking lipid peroxidation^81^. Activation^41^ and up-regulation^82^ of gene expression of enzymatic antioxidants and scavenging free radical^83^ by thymoquinone could underlie the suppression in lipid peroxidation and the enhancement in TAC in our experimental model. Squalene enriched-herbal interventions show a promising dual antioxidant ability both by enhancing antioxidant activities and quenching the reactive oxidants^84^. Squalene sprayed in the bedding material increased the activities of glutathione peroxidase, catalase and SOD when concurrently administrated with 3‐methylcholanthrene in rats^85^. Boosting the activity of components of glutathione redox cycle and reducing the activity of xanthine oxidase by farnesol play a central role against cigarette smoke extract-induced oxidative stress in the prostate of rats^86^. Again, the cytological modifications found in our study were in accordance to the antioxidant effects of NSS; e.g., the immuno-expression of GR and SOD2 in the spermatogenic and Leydig cells confirmed the reduction in TAC following dietary inclusion of NSS.

The histopathological lesions in testicular tissues exposed to MSG is similar to those observed by other investigators^49^. The depopulation of germ cells due to inhibition of cell division is responsible for atrophy of ST^87^. The degenerative changes in germ and somatic cells of ST may be arisen from the interaction of MSG with proteins and enzymes interfering with the antioxidant defense mechanism leading to accumulation of free radicals which in turn induces inflammatory reaction and mitochondrial damage^88^. On the contrary, other studies did not find any histological lesions in the testicular tissue of animals received MSG^10,89^ due to intake of low doses.

In our study, ST in MSG group were characterized by reduction in the thickness of basement membrane, diameter and height of its epithelial lining, and number of cellular layers in parallel with the degeneration in Sertoli cells and germinal epithelium. These morphometric changes are in consistent with previous studies on the cytotoxic effects of MSG on the testes of rat^49,90^. The congestion in the blood vessels might be due to suppression in prostaglandins synthesis, since these bioactive molecules are known to be involved in the regulation of testicular blood flow^91^. The adverse alterations in the histological features of testis could be explained by both direct and indirect mechanistic pathways. The direct effect is supposed to be mediated by glutamate receptors and transporters that are expressed in some effector sites in the testicular microenvironment^92,93^. The indirect effects involve disruption of the hypothalamic–pituitary–gonadal axis, that control the rate of spermatogenesis and secretion of testosterone, culminating at structural and functional modifications in the testicular tissue^49^. The other indirect effect is exhaustion of the testicular ascorbic acid reserve following MSG intoxication leading to oxidative damage in the testes of rat^94^.

The irregular and interrupted arrangement pattern in the collagen fibers around the ST in MSG group is in consistent with the ability of MSG to deposit excess collagen fibers in intertubular interstitial tissue and perivascular areas^49^ and induce renal interstitial fibrosis^95^. The reactive free radical overgeneration following MSG intake is suggested to drive fibroblast-to-myofibroblast differentiation^96^.

The remarkable improvement in the testicular histo-architecture of MSG challenged rats following NSS administration is in harmony with other researchers^97^. The active phytochemical compounds of NSS as thymoquinone, thymol and α-hederin are central testicular protectants against harmful agents by inhibition of iron-related lipid peroxidation, nuclear factor kappa B, cyclooxygenase and lipoxygenase, and elevation of enzymatic and non-enzymatic components of antioxidant network^98^. Hormonal rebalance in the reproductive axis and antioxidant and anti-apoptotic properties of thymoquinone along with the ability of NS to act as a vasodilator to the microvasculature^99^ could be implicated in reestablishing the testicular histological patterns and protecting it from degenerative and necrotizing chemo-toxicants. The stimulatory effects of NSS on spermatogenesis^15^ results in preservation of the spermatogenic cells in different stages of development up to mature sperm. The improvement in the testicular histomorphometric indices in the current study is similar to that found in heat stressed^100^ and chlorpyrifos challenged testis^16^. The ability of NSS to protects cell proliferation leads to enhanced regeneration after tissue damage^101^.

SODs are family of important enzymatic antioxidants that protect the cells against superoxide toxicity^102^. GR is fundamental in preserving the cellular content of reduced glutathione; one of the most abundant reducing thiols which plays a crucial role in the regulation of reactive oxygen species generation^103^. Based on our findings, it seemed that oxidative stress caused by MSG exposure induced free radical scavengers such as SOD2 and GR to protect the cells from the injury by reactive oxygen species.

The low expression of SOD2 and GR in the germinal and Leydig cells in MSG + NSS group is in contrast to the up-regulation of enzymatic antioxidants in the liver of hypercholesterolemic rats^82^ and cortex and hippocampus of lead challenged mouse^104^. This conflict data may be attributed to differences in the experimental animal models. The weak immunostaining of SOD2 and GR reflects the free radical scavenging activity of NSS^104^.

The pro-apoptotic activity of MSG, manifested by up-regulation in immuno-expression of caspase 3 in the testicular tissues, is compatible with the findings of previous studies^1,49^. Presence of excessive amount of glutamate secondary to MSG intake is implicated in overactivation of glutamate receptors producing intracellular calcium waves^105^. These in turn lead to activation of calcium-dependent caspases and terminate at apoptosis^106^. Induction of oxidative stress by MSG may be considered as another causative factor in prompting cell death by triggering extrinsic and intrinsic apoptotic pathways^49,107^. In agreement with a previous study^81^, concurrent administration of NSS to MSG intoxicated rats provided cytoprotection against the development of MSG-induced apoptosis which can be explained based on its antioxidant and anti-inflammatory properties^108^. Thymoquinone exhibits anti-apoptotic properties evident by down-regulation in immuno-expression of caspase 3 and heat shock proteins in the testis of doxorubicin challenged rats^109^. Stabilization of the cell membrane due to its hydrophobicity^110^ and inhibition of ataxia telangiectasia mutated kinase-dependent signaling pathway^111^ are possible mechanistic pathways by which squalene blocks the action of pro-apoptotic inducers. Linolenic acid (octadecadienoic acid) rescued Bcl-2 expression, inhibited Bax translocation to mitochondria and suppressed caspase-3 activity in Murine C2C12 myoblasts^112^.

In conclusion, this study illustrated the protective effect of NSS against structural and functional testicular deteriorations associated with MSG intake through restoring the hormonal balance of pituitary–gonadal axis, enhancing redox homeostasis, and exerting anti-apoptotic effect. These results are of outmost significance in paving the road towards incorporation of NSS as a spice and food preservative in our food industry as well as a health remedy in the traditional medicine to fight the MSG-related reproductive abnormalities and giving a strong driving force for exploring its other potential mechanistic avenues.
